# Comparison of mean operative time in patients undergoing Ho: YAG laser lithotripsy and pneumatic lithotripsy in ureterorenoscopy for ureteric calculus

**DOI:** 10.12669/pjms.37.2.3049

**Published:** 2021

**Authors:** Muhammad Tanveer Sajid, Mohammad Ameen, Badar Murtaza, Muhammad Sarwar Alvi, Zakir Khan, Faran Kiani

**Affiliations:** 1 Muhammad Tanveer Sajid, Armed Forces Institute of Urology (AFIU), Rawalpindi, Pakistan; 2 Mohammad Ameen, Armed Forces Institute of Urology (AFIU), Rawalpindi, Pakistan; 3 Badar Murtaza, Armed Forces Institute of Urology (AFIU), Rawalpindi, Pakistan; 4 Muhammad Sarwar Alvi, Armed Forces Institute of Urology (AFIU), Rawalpindi, Pakistan; 5 Zakir Khan, Armed Forces Institute of Urology (AFIU), Rawalpindi, Pakistan; 6 Faran Kiani, Armed Forces Institute of Urology (AFIU), Rawalpindi, Pakistan

**Keywords:** Laser lithotripsy, Lasers Solid state, Operative time, Pneumatic lithotripsy, Ureteral calculi, yttrium-aluminum-garnet

## Abstract

**Objective:**

To compare the mean operative time (MOT) in patients undergoing Ho: YAG laser lithotripsy (LL) and pneumatic lithotripsy (PL) for ureteric stones.

**Methods:**

This randomized study was conducted at Armed Forces Institute of Urology (AFIU) Rawalpindi, Pakistan from July 2016 to November 2018. Non probability consecutive sampling technique utilized to enroll 60 patients of both gender aged 18-60 years, having ureteric calculus ≤1.5cm. Randomization was done into group I (LL) and II (PL) via computer generated number tables. Six Consultant Urologists performed surgeries under spinal anesthesia utilizing Swiss Lithoclast^®^ Master (EMS^+^ S.A. Switzerland) in group II and holmium laser fiber (365μm, 8-10Hz, 9.6-16W, 2100nm wavelength) in group I respectively. MOT was noted from insertion of cystoscope till removal out of meatus. Data obtained was analyzed through IBM SPSS 24.0.

**Results:**

Analysis involved 60 patients (30 each group) having similar baseline characteristics (age, gender, laterality, location). There was statistically significant different MOT between LL & PL (25.48±6.99 vs 34.83± 7.47 minutes, p < 0.001). Data stratification with respect to age, gender, laterality and stone location revealed similar trend. Lithotripsy technique significantly affected MOT (p < 0.001) on Multiple Linear Regression Analysis.

**Conclusions:**

Ho: YAG LL is an efficient technique when compared with PL in terms of MOT for ureteric stones.

## INTRODUCTION

Urolithiasis, a highly recurrent disease, is affecting 15% of world population having serious implications as rapidly increasing obesity; diabetes and western lifestyle are exponentially compounding the issue which has no cure.^1^ Middle East is having highest life time risk (50% at 5 years & 70% at 9) while Pakistan being part of Afro-Asian stone region has prevalence of 4-20%.^2^ A complex interplay of intrinsic as well as extrinsic factors over background of genetic and anatomical characteristics leads to stone formation. Urinary calculi can be classified on the basis of size, location, radiological features, etiopathogenesis, composition, and risk of recurrence.^3^

Males are affected more, peak decades of presentation being 3^rd^ and 4^th^_._ Symptoms depend upon location; most common are acute flank pain and hematuria. Optimal treatment and prevention depends upon clinical, anatomical, technical and stone factors.^4^ Past couple of decades has witnessed paradigm shift from open surgery to extracorporeal shock wave lithotripsy (ESWL), ureterorenoscopy (URS), laparoscopic ureterolithotomy and percutaneous nephrolithotomy (PCNL).^5^ Endoscopy is the treatment of choice worldwide due to miniaturization of equipment and availability of wide array of intracorporeal lithotripters (electrohydraulic, ultrasonic, pneumatic and laser). Most common lithotripter currently in use are pneumatic (PL) and holmium: yttrium-aluminum-garnet (Ho: YAG) laser (LL), the latter recommended by European association of urology (EAU) as gold standard.^6^

PL, introduced in 1992 in Switzerland, is a favored technique due to easy installation, safety, cost effectiveness, wide availability and short learning curve but at the cost of higher stone migration and inability of its use with flexible URS.^7^ Ho: YAG LL is most efficient and versatile tool due to its ability to break stones independent of composition, lower risk of stone migration, higher stone free rates and minimal stricture formation.^8^ Downside includes high price, long learning curve and availability.^9^ The literature so far in our country is limited and inconclusive regarding both techniques in terms of operative time, stone fragmentation and stone free rate.^10^ We aimed to determine MOT of both in our setting thus anticipating time slots available and manage operation list in a better way.

## METHODS

Current study was conducted at AFIU, Rawalpindi over 02 years after approval by the hospital Ethical Review Board (ERB) (Certificate no Uro-Adm-Trg-1/IRB/2016/105). The sample size was calculated in the light of literature.

### SAMPLE SIZE:

Sample size was calculated by using the WHO calculator utilizing data from study by Linjin L *et al*. 17

Level of significance = 0.05 or 5%

Power of test = 80%

Population mean = 10.8

Test value of population mean = 28 17

Anticipated population mean = 41 17

Sample size = 60 (30 patients in each group)

Non probability consecutive sampling technique utilized to enroll 60 patients of both genders aged 18-60 years, having ureteric calculus (≤1.5cms) confirmed on Computed tomography Kidney, ureter, and bladder (CT KUB). Patients having congenital renal anomalies, previous history of ureteric intervention, spinal deformities and bleeding diathesis were excluded. Radom generated computer number tables were used to divide study population into Group-I (LL) and Group-II (PL). Written informed consent obtained and demographic details noted.

Six consultant urologists performed surgeries under spinal anesthesia after administration of prophylactic antibiotics. Rigid cystoscopy was performed to locate ureteric orifice while 6-7.5 F semi-rigid URS (Karl Storz) utilized to advance guide wire (0.035 inch, Boston scientific TM Guide, USA) under vision. Swiss Lithoclast^®^ Master (EMS^+^ S.A. Switzerland) (0.8 mm or 1 mm probe) was used to break calculi in group II after placement of cone (Stone Cone™ Nitinol Retrieval Coil, Boston Scientific USA). In group I, the holmium laser fiber (365μm) pulse frequency: 8-10Hz and power supply: 9.6-16W with 2100 wavelength (nm) and ≈0.5 mm tissue penetration characteristics was utilized. Large stone fragments retrieved while those <3 mm were left for spontaneous passage. A 4.5F double J stent was placed in all and removed 02 weeks post operatively. Foley catheter was removed on 1^st^ post-operative day. Operation time was noted from insertion of cystoscope till the removal of uretrorenoscope (URS) out of meatus.

Data obtained was analyzed through IBM SPSS Statistics for Windows, Version 24.0. (Armonk, NY: IBM Corp).^11^ Frequencies and percentages were calculated for categorical variables (gender, age groups, stone location and side of stones) and Chi-Square test used for inference statistics while mean ± SD was calculated for continuous variables (age, MOT; normally distributed as revealed by Kolmogorov-Smirnov test) and Independent Samples t-test applied. MOT among different age groups and stone locations was compared by One-Way Analysis of Variance (ANOVA) test. The Multiple Linear Regression Analysis model was employed to measure the association between the types of lithotripsy technique and MOT after controlling the possible confounders; age, gender, side of stone and location of stone. P-value <0.05 was considered significant.

## RESULTS

Sixty patients majority being male (49 vs 11 female) were studied. Baseline characteristics (age, gender, stone laterality, location) were comparable in both groups (statistically insignificant) (Table-I). MOT revealed no statistically significant difference with respect to age groups, gender, laterality and stone location (p > 0.05, One-Way ANOVA; Table-II).

**Table-I:** Demographic variables of the patients included in the study (n=60).

*Demographic variable*	*Type of Intra-ureteral Lithotripsy*		*p value*

	*Group I (n=30)(LL)*	*Group II (n=30)(PL)*	
Age (years) Mean ± SD	35.00± 12.59	38.80± 11.51	0.207
Gender Male: Female n / %	23(76.7%):7(23.3%)	26(86.7%):4(13.3%)	0.317
***Age groups (Years)***			
18 - 23	5(16.7%)	2(6.7%)	
24 - 29	8(26.7%)	5 (16.7%)	
30 - 35	5(16.7%)	7(23.3%)	
36 - 41	3(10%)	5(16.7%)	
42 - 47	3(10%)	3(10%)	
48 - 53	2(6.7%)	4(13.3%)	
54 - 60	4(13.3%)	4(13.3%)	
***Side of Stone***			
Right	15(50%)	15(50%)	1.00
Left	15(50%)	15(50%)	
***Stone Location***			
Proximal Ureter	13 (43.3%)	12 (40%)	
Mid Ureter	09(30%)	08(26.7%)	0.852
Lower Ureter	08(26.7%)	10 (33.3%)	
***Stone Characteristics***			
Right Lower	3 (10%)	5(16.7%)	
Right Mid	4(13.3%)	4(13.3%)	
Right Upper	8(26.7%)	6(20%)	0.724
Left lower	5(16.7%)	5(16.7%)	
Left Mid	5(16.7%)	4(13.3%)	
Left Upper	5(16.7%)	6(20%)	

**Table-II:** Mean operation time of study population with respect to demographic variables (n=60).

*Demographic variable*	*n*	*Operation time (Mean ± SD) (Minutes)*	*t-value (df)*	*p value*
***Age groups (Years)***				
18 - 23	7	28.14 ± 11.8	0.561(6, 53)	*0.759
24 - 29	13	27.96 ± 9.05		
30 - 35	12	33.42 ± 10.4		
36 - 41	8	29.81 ± 6.03		
42 - 47	6	32.00 ± 7.07		
48 - 53	6	28.50 ± 5.62		
54 - 60	8	30.81 ± 7.71		
***Gender***				
Male	49	31.06 ± 8.59	1.75 (58)	0.086
Female	11	26.14 ± 7.69		
***Laterality***				
Right	30	29.45 ± 9.32	-0.64 (58)	0.527
left	30	30.87 ± 7.88		
***Stone location***				
Proximal ureter	25	29.10 ± 8.42	0.320 (2,57)	*0.728
Mid Ureter	17	31.00 ± 9.12		
Distal Ureter	18	30.83 ± 8.64		

* One-Way ANOVA

Statistically significant different MOT was found between LL and PL group (25.48±6.99 vs 34.83± 7.47) (p < 0.001, Independent Samples t-test). Similar trend was noted when data was stratified with respect to age groups, gender, laterality and stone location except age group 18-23,48-53,54-60 and mid-ureteric stone location (Table-III). The Multiple Linear Regression Analysis performed revealed MOT to be significantly higher in PL group (9.13 minutes more) after controlling all other variables (age, gender, laterality and location of stones) of the model (p < 0.001) (Table-IV).

**Table-III:** Mean operative time in both groups baseline and stratified with respect to age groups, Gender, laterality, stone location (n=60).

*Variable*	*Group I (n=30)(LL)*		*Group II (n=30)(PL)*		*t-value (df)*	*P value*
	
	*(Mean ± SD)*	*95% C.I*		*(Mean ± SD)*			*95% C.I*	
	
		*Lower bound*	*Upper bound*				*Lower bound*	*Upper bound*
Operative Time (minutes)	25.48 ± 6.99	22.87	28.09	34.83 ± 7.47	32.04	37.62	-5.00(58)	< 0.001
	
	*Operative Time (minutes) (Mean ± SD)*			*Operative Time (minutes)(Mean ±SD)*				
***Age groups (Years)***						
18 - 23	25.30 ± 11.0			35.25 ± 14.5			- 1.01(5)	0.36
24 - 29	23.87 ± 6.49			34.50 ± 9.23			- 2.45(11)	0.03
30 - 35	26.70 ± 10.0			38.21 ± 8.18			- 2.19(10)	0.053
36 - 41	25.00 ± 4.50			32.70 ± 5.07			- 2.16 (6)	0.07
42 - 47	25.83 ± 0.76			38.17 ± 3.21			-6.47(4)	0.003
48 - 53	27.00 ± 4.95			29.25 ± 6.50			- 0.42(4)	0.69
54 - 60	25.48 ± 6.99			34.88 ± 7.19			- 1.67(6)	0.15
***Gender***								
Male	26.61 ± 7.22			35.00 ± 7.83			- 3.89 (47)	<0.001
Female	21.79 ± 4.97			33.75 ± 5.14			- 3.79 (9)	0.004
***Laterality***								
Right	23.67 ± 7.59			35.23 ± 7.11			- 4.31 (28)	<0.001
Left	27.30 ± 6.05			34.43 ± 8.05			- 2.74 (28)	0.01
***Stone location***								
Proximal ureter	24.35 ± 5.65			34.25 ± 8.02			- 3.59 (23)	0.002
Mid Ureter	27.78 ± 9.77			34.63 ± 7.26			- 1.62 (15)	0.13
Distal Ureter	24.75 ± 5.41			35.70 ± 7.68			- 3.40(16)	0.004

Student t-test

**Table-IV:** Multiple Linear Regression analyses of factors associated with the mean operative time taken for the intra-ureteral lithotripsy procedure (n=60).

*Independent Variables*	*B*	*S.E*	*t*	*p-value*
Type of Intra-ureteral lithotripsy	9.13	1.93	4.72	0.000
Age of Patient	-0.035	0.08	-0.42	0.674
Gender of Patient	-3.33	2.52	-1.32	0.191
Stone Location	0.16	1.17	0.14	0.893
Side of Stone	1.44	1.94	0.74	0.462

## DISCUSSION

A large population worldwide is suffering from crystalline deposits formed due to the deposition of phosphates, calcium, and oxalates called urinary calculi.^12^ Optimal treatment modality is dictated by clinical, technical, stone and patient factors. Current urological practices witnessed paradigm strides from open surgery, the endoscopy evolving as treatment of choice for ureteral stones.^13^ Most frequent lithotripter in use currently are PL and LL, the latter recommended by EAU as gold standard.^14^ This study evaluated the effect of both in terms of MOT in the treatment of ureteral calculi.

Our results are coherent with studies conducted locally as well as internationally. Yin *et al*.^15^, in their meta-analysis which included four trials and 295 patients, showed significant benefits of LL in terms of MOT as compared to PL (p value < 0.001). Similar findings were observed by Demir *et al*.^16^ They concluded that the usage of LL in patients with ureteral stones is more effective than PL in terms of operation time (15.25 ± 6.14 vs 33.05 ± 11.36, *p value* < 0.05). Another study by Linjin *et al*.^17^ also confirmed less operating time with LL (28 ± 9.2 vs 41 ± 12.4, *p* 0.001) as suggested by our study. Zyczkowski M *et al*.^18^ studied 108 children and found statistically significant shorter MOT in LL group (34 vs 56 minutes, p 0.04).

However, the results published by Tipu *et al*.^10^ contradict our findings. Although MOT in both groups was found similar (39.6 ± 11.9 vs37.2 ± 13.0, p< 0.07), the stone free rate was better in LL group. Studies conducted by Degirmenci *et al*.^19^, Abedi AR et al.^20^ and Razzaghi *et al*.^21^ found PL to be more time efficient in comparison to LL {(28.4 ± 9.7 vs 32.2 ± 11.1, *p* 0.035) (10.01 ± 6.2 vs 14.4 ± 2.05, p ≤0.05) & (7.9 ± 4.2 vs 13.7 ± 12.6, *p* 0.029) respectively}. Same notion was supported by Akdeniz *et al*.^22^ Baseline characteristics of all these above mentioned studies are comparable to our data. In the current study, in patients having proximal and lower ureteric stones, the MOT was significantly different between LL & PL group (p < 0.05). However, it was insignificant in case of mid ureteric stones (p > 0.05). Khoder et al.^23^ reported statistically significant MOT between proximal and distal ureteric stones in LL group contrary to our findings (81.3 ± 4.5 vs 65.7 ± 3.8, *p* 0.017).

A recent meta-analysis conducted by Chen S *et al*.^24^ analyzing eight studies having 1,555 patients (weighted mean difference = –11.52, 95% CI –17.06 to –5.99, p < 0.0001), conferred to our study, concluding that further additional trails are unlikely to alter their results. Similar supporting conclusions were made by Kadihasanoglu M et al.^25^ in their trial. Thus, the overall picture favors LL as treatment of choice for ureteral stones as far as MOT is concerned.

### Limitations of the study

The results of present study should be interpreted with caution as it involved only sixty subjects from single center and short follow up. Procedure was performed by six surgeons, which might have led to potential bias in MOT. Moreover, stone free rate, complication rate and stricture formation rate was not studied thus missing important aspect of modality judgment.

## CONCLUSIONS

Ho: YAG LL is an efficient technique when compared with PL in terms of MOT helping us manage long operation list in a better way saving precious time in already overwhelmed healthcare system. We recommend more high quality, multicenter RCTs with long term follow up encompassing various aspects to better assess superiority of LL.

### Authors’ Contribution:

**MTS, MA:** Conceived, designed and did statistical analysis & editing of manuscript

**MTS, MA, ZK:** Did data collection

**BM, MSA, FK:** Reviewed, provided technical support and final approval of manuscript

All authors of this paper have equally contributed to this study and approved the final version to be published and are responsible for integrity of the research.
